# Serum Carnosinase-1 and Albuminuria Rather than the *CNDP1* Genotype Correlate with Urinary Carnosinase-1 in Diabetic and Nondiabetic Patients with Chronic Kidney Disease

**DOI:** 10.1155/2019/6850628

**Published:** 2019-12-24

**Authors:** Angelica Rodriguez-Niño, Sibylle J. Hauske, Anna Herold, Jiedong Qiu, Jacob van den Born, Stephan J. L. Bakker, Bernhard K. Krämer, Benito A. Yard

**Affiliations:** ^1^Vth Department of Medicine (Nephrology/Endocrinology/Rheumatology), University Medical Center Mannheim, University of Heidelberg, Mannheim 68167, Germany; ^2^Department of Nephrology, University Medical Centre Groningen, University of Groningen, Groningen 9700RB, Netherlands

## Abstract

**Background:**

Carnosinase-1 (CN-1) can be detected in 24 h urine of healthy individuals and patients with type 2 diabetes (T2DM). We aimed to assess whether urinary CN-1 is also reliably measured in spot urine and investigated its association with renal function and the albumin/creatinine ratio (ACR). We also assessed associations between the *CNDP1* (CTG)*_n_* genotype and CN-1 concentrations in serum and urine.

**Methods:**

Patients with T2DM (*n* = 85) and nondiabetic patients with chronic kidney disease (CKD) (*n* = 26) stratified by albuminuria (ACR ≤ 300 mg/g or ACR > 300 mg/g) recruited from the nephrology clinic and healthy subjects (*n* = 24) were studied.

**Results:**

Urinary CN-1 was more frequently detected and displayed higher concentrations in patients with ACR > 300 mg/g as compared to those with ACR ≤ 300 mg/g irrespective of the baseline disease (T2DM: 554 ng/ml [IQR 212-934 ng/ml] vs. 31 ng/ml [IQR 31-63 ng/ml] (*p* < 0.0001) and nondiabetic CKD: 197 ng/ml [IQR 112-739] vs. 31 ng/ml [IQR 31-226 ng/ml] (*p* = 0.015)). A positive correlation between urinary CN-1 and ACR was found (*r* = 0.68, *p* < 0.0001). Multivariate linear regression analysis revealed that ACR and serum CN-1 concentrations but not eGFR or the CNDP1 genotype are independent predictors of urinary CN-1, explaining 47% of variation of urinary CN-1 concentrations (*R*^2^ = 0.47, *p* < 0.0001).

**Conclusion:**

These results confirm and extend previous findings on urinary CN-1 concentrations, suggesting that assessment of CN-1 in spot urine is as reliable as in 24 h urine and may indicate that urinary CN-1 in macroalbuminuric patients is primarily serum-derived and not locally produced.

## 1. Introduction

Diabetic nephropathy (DN) is considered to be one of the most devastating microvascular complications of diabetes mellitus (DM), developing in nearly one-third of patients with type 1 or type 2 diabetes [1]. It is by far the most common cause of chronic kidney disease (CKD) worldwide, frequently leading to end-stage renal disease (ESRD) and the need for renal replacement therapy [2]. The combination of ESRD and diabetes also imparts a hugely increased risk of cardiovascular events [3, 4] and mortality [5].

DN typically develops through sequential phases, starting with hyperfiltration, followed by the onset of albuminuria and progressive decline in the glomerular filtration rate (GFR) [6, 7]. Both eGFR and albuminuria are independent risk factors for mortality and progression to ESRD and display a strong synergy in increasing risk [8]. There is compelling evidence indicating that susceptibility to developing DN is, at least in part, genetically determined [9]. Amongst the reported susceptibility loci, we have repeatedly reported in the last decade on genetic variants of the *CNDP1* gene that are associated with serum carnosinase-1 (CN-1) levels and the development of DN.

CN-1 is encoded by the *CNDP1* gene, which harbours a trinucleotide length (CTG)*_n_* polymorphism in the signal peptide of CN-1 that influences secretion of the CN-1 protein [10]. We and others have demonstrated that the *CNDP1* (CTG)*_n_* polymorphism is associated with susceptibility to developing DN in T2DM [11–15]. The shortest allelic form, i.e., the *CNDP1* (CTG)_5_ or Mannheim allele, is more common in the absence of nephropathy and is associated with low CN-1 enzymatic activities and low serum concentrations [10, 11, 16]. Nonetheless, it should also be mentioned that other studies failed to demonstrate this association in cohorts of different ethnicities [17, 18]. In patients with type 1 diabetes, there are also inconsistent findings related to the association between *CNDP1* and ESRD. While in a genome-wide SNP genotyping approach in 1,906 unrelated Caucasian individuals with type 1 diabetes, an association between ESRD and *CNDP1* was observed [19], this was not found in another case-control study, consisting of 1,269 Caucasian patients with type 1 diabetes [20]. Likewise, Alkhalaf et al. did not find an association between the homozygous *CNDP1* (CTG)_5_ genotype and DN in patients with type 1 diabetes but rather an increased risk of progression to ESRD late after baseline measurements [21].

The influence of the *CNDP1* Mannheim allele has also been investigated in nondiabetic nephropathies, where it was found to be associated with a slower progression to CKD and to correlate with renal survival in patients with glomerulopathies, but not in patients with tubulointerstitial disease [22, 23]. Notwithstanding the foregoing, there is compelling evidence that carnosine, a major substrate of CN-1, has renoprotective properties in animal models of diabetes [24–27]. Likewise, in human studies in patients with T2DM, carnosine supplementation has been shown to have a beneficial effect on hyperglycaemia [28, 29], triglycerides, and inflammatory mediators [29].

The recent finding that the human kidney possesses an intrinsic carnosine metabolism [30] and that CN-1 is detectable in urine of healthy subjects and patients with T2DM [31] underscores its biological relevance in the context of kidney disease. While initial studies have shown an increased expression of CN-1 in biopsies of patients with DN [11, 30], we more recently demonstrated that CN-1 concentrations in 24 h urine samples are increased in patients with T2DM and macroalbuminuria and that this correlates with urinary albumin excretion and renal function [31]. Although 24 h urine collection has been considered to be the gold standard for the assessment of albuminuria, specimens of the first void or spot urine rather than 24 h urine are more commonly collected in biorepositories of patients with CKD.

Spot urine however is liable to hourly variations in urinary protein and creatinine excretion and therefore may deviate from 24 h urine ACR assessment. Hence, to confirm our previous findings in larger cohorts where 24 h urine collections were available, the present study was conducted as a proof of principle and sought to assess the relation between CN-1 in spot urine, ACR, and renal function and to replicate our earlier findings with 24 h urine collections. In essence, the current study addressed (1) whether CN-1 can be reliably measured in spot urine, (2) whether there is an association between urinary CN-1, eGFR, and albuminuria in diabetic and nondiabetic patients with chronic kidney disease, (3) whether high urinary CN-1 concentrations in macroalbuminuric patients are associated with low CN-1 concentrations in serum, and (4) whether the (CTG)*_n_* polymorphism known to determine CN-1 secretion is associated with urinary CN-1 concentrations.

## 2. Materials and Methods

### 2.1. Participants and Sampling

#### 2.1.1. Participants

A total of 111 patients (i.e., patients with T2DM and chronic kidney disease (*n* = 85) and nondiabetic patients with other causes of CKD (*n* = 26)) were included in this study and stratified on the basis of the albumin/creatinine ratio (ACR) in macroalbuminuria (ACR > 300 mg/g) and micro- or normoalbuminuria (ACR ≤ 300 mg/g) groups. For patient allocation, at least 2 independent assessments with persistent ACR findings were required. Demographic and clinical characteristics of the groups are presented in Table 1.

Diabetes mellitus was defined by a documented history of diabetes or a fasting blood glucose ≥ 7.0 mmol/l (126 mg/dl), a casual plasma glucose level ≥ 11.1 mmol/l (200 mg/dl), or HbA1c ≥ 6.5% (48 mmol/mol). Nondiabetic patients with CKD were included after screening of patient medical records, medications, and laboratory testing for plasma glucose or HbA1c to exclude diabetes.

Parameters including glycated hemoglobin (HbA1c), body mass index (BMI), serum creatinine, and albuminuria/creatinine ratio (ACR) were extracted from medical records. The estimated glomerular filtration rate (eGFR) was calculated according to the CKD-EPI formula. Patients that underwent kidney transplantation or patients without residual diuresis were excluded. Subjects with missing urine and blood sampling were also excluded. The study was approved by the local ethics committee, and all patients gave written informed consent prior to the study enrolment (no. 0193/2001). The healthy controls consisted of 24 adults (16 females and 8 males) recruited from our laboratory staff that voluntarily decided to participate, and all gave written informed consent to study enrolment. Healthy controls had no history of diabetes and cardiovascular or kidney disease.

#### 2.1.2. Sampling

Serum and spot urine samples were collected to assess CN-1 concentration. Genotyping was performed on EDTA blood. All samples were stored at −20°C until use.

### 2.2. *CNDP1* Genotyping

Genomic DNA was isolated from EDTA blood using the Genomic DNA Isolation Kit (Promega, Mannheim, Germany). Thereafter, a standard PCR protocol was used with the fluorescence-labeled forward primer 5′-FAM-GCGGGGAGGGTGAGGAGAAC-3′ and the unlabeled reverse primer 5′GGTAACAGACCTTCTTGAGGAATTTGG-3. After PCR amplification, fragment analysis was performed with an ABI310 analyzer (PerkinElmer) to determine the fragment length corresponding to the different genotypes. Each peak corresponded with the number of leucine repeats on each allele. The 157, 160, and 163 bp products corresponded with the (CTG)_5_, (CTG)_6_, and (CTG)_7_ alleles encoding for five, six, and seven leucine repeats, respectively.

### 2.3. Serum and Urinary CN-1 Concentrations

CN-1 concentrations in serum and urine were measured by ELISA as previously described by Adelmann et al. [32] and Rodriguez-Niño et al. [31], respectively. In brief, high-absorbent microtiter plates (Greiner, Labortechnik, Frickenhausen, Germany) were coated overnight with 100 *μ*l of goat polyclonal anti-human CN-1 (10 *μ*g/ml) (R&D, Wiesbaden, Germany). The plates were extensively washed and incubated with 0.05% of dry milk powder to avoid unspecific binding. Serum samples were tested in a dilution of 1 : 100 or 1 : 200, and urine samples were tested undiluted. The plates were placed on a shaker for 1 hour and subsequently extensively washed with 1x PBS/Tween. Thereafter, a rabbit polyclonal antibody (ATLAS, Abcam plc, Cambridge, United Kingdom) was added for 1 hour followed by extensive washing. A goat anti-rabbit IgG HRP-conjugated antibody was added for 30 min followed by extensive washing. Deep-blue peroxidase (POD) (Roche Diagnostics, Mannheim, Germany) was used for color development which was stopped after 20 min by the addition of 50 *μ*l of 1 M H_2_SO_4_. The plates were directly read at 450 nm fluorescence. A serial dilution of pooled serum with a known carnosinase concentration (2 *μ*g/*μ*l) was used as the standard. CN-1 protein concentrations were assessed in the linear part of the dilution curve with a lower detection limit of 31 ng/ml. Concentrations below the detection limit were set at 31 ng/ml.

### 2.4. Statistical Analysis

Quantitative data were expressed as means ± SD or SEM or median with the corresponding interquartile range (IQR). For comparison of the groups, independent Student's *t*-test was applied for data with normal distribution or the Mann-Whitney *U* or Kruskal-Wallis test for data with nonnormal distribution. Qualitative data were expressed as numbers and percentages and analyzed using the *χ*^2^ test or Fisher exact test with Bonferroni correction when appropriate. The correlations between urinary CN-1 and other parameters were analyzed by the Pearson or Spearman correlation analysis. Due to a skewed distribution, urinary CN-1 concentrations were logarithmically transformed together with the variables ACR and serum CN-1: log_10_ (urinary CN-1), log_10_ (ACR), and log_10_ (serum CN-1). Variables with a *p* value < 0.25 in the univariate analysis were included in the multivariate model. Multivariate regression analysis was employed with log-transformed urinary CN-1 as the dependent variable to determine the best multivariate model predicting urinary CN-1 concentrations. The association of serum CN-1 (log_10_-transformed serum CN-1) with kidney disease was analyzed with linear regression analysis with adjustment for age and sex (model 1) and urinary CN-1 (model 2). All statistical tests were two-sided, and a *p* value < 0.05 was considered statistically significant in all analyses. The analyses were assessed with GraphPad Prism 7.02 (GraphPad Software, Inc., La Jolla, California) and SPSS 23.0 software (SPSS, Inc., Chicago, IL).

## 3. Results

### 3.1. Demographic and Clinical Characteristics

CN-1 concentrations in serum and in spot urine as well as the *CNDP1* genotype were assessed in patients with T2DM (*n* = 85), in patients with CKD but no diabetes (*n* = 26), and in healthy controls (*n* = 24). T2DM patients were further stratified on the basis of ACR into patients with normo- or microalbuminuria (ACR ≤ 300 mg/g) (*n* = 44) and patients with macroalbuminuria (ACR > 300 mg/g) (*n* = 41). Likewise, patients with nondiabetic CKD were stratified into the same groups (ACR ≤ 300 mg/g) (*n* = 9) (ACR > 300 mg/g) (*n* = 17).

Importantly, in the overall patient cohort (*n* = 111), only 18% (20 of 111) of the patients had normoalbuminuria (ACR < 30 mg/g) and less than 14% (16 of 111) of the patients showed an eGFR above 60 ml/min/1.73m^2^ reflecting a population with predominantly severe renal function impairment. Although 41% of all patients (46 of 111) were on renal replacement therapy (hemodialysis or peritoneal dialysis), all had residual urine output.

All relevant demographic and clinical characteristics of the groups are depicted in Table 1. The healthy control group consisted of adults without clinical signs or medical history of renal disease or diabetes. In the control group, there were more females included and the mean age was significantly lower as compared to both disease groups (*p* < 0.0001). No differences in this respect were observed between the T2DM and CKD groups as a whole or between the ACR subgroups of T2DM and CKD, respectively. As expected, T2DM patients with ACR > 300 mg/g displayed more frequently retinopathy and had higher serum creatinine levels and lower eGFR compared to T2DM patients with ACR ≤ 300 mg/g. Macroalbuminuric patients in the CKD group displayed trends for increased serum creatinine and lower eGFR in comparison to CKD patients with normo- or microalbuminuria, but these differences did not reach statistical significance. The main causes of CKD of nondiabetic aetiology were IgA nephropathy, focal glomerular sclerosis, and other glomerulonephritides.

### 3.2. Urinary CN-1 Concentrations Are Increased in Patients with Macroalbuminuria

**Figure 1:**
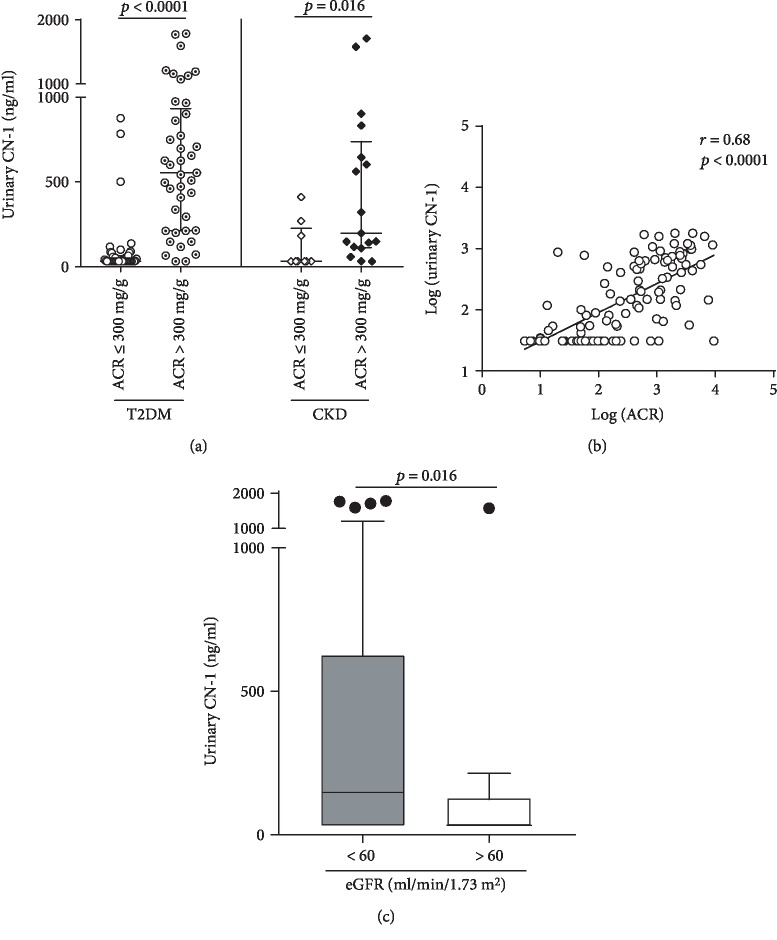
(a) Distribution of CN-1 in spot urine of T2DM and nondiabetic patients (CKD) with normo- or microalbuminuria (ACR ≤ 300 mg/g) and macroalbuminuria (ACR > 300 mg/g). Results for each individual patient are shown as well as the median and IQR (lines) for each of the different groups. (b) Pearson correlation between ACR [log (ACR)] and urinary CN-1 concentrations [log (urinary CN-1)] in patients with T2DM and nondiabetic CKD patients (*n* = 111). (c) Distribution of urinary CN-1 concentrations in all patients according to eGFR. T2DM and CKD patients (*n* = 111) were stratified according to reduced (eGFR < 60 ml/min/1.73m^2^) (*n* = 95) and preserved renal function (eGFR > 60 ml/min/1.73m^2^) (*n* = 16). eGFR was estimated using the CKD-EPI formula. Boxes and whiskers represent the median and IQR. Outliers are indicated by circles.

In the group of patients with T2DM and macroalbuminuria, CN-1 was detected in 95% (39 of 41) of the urine samples while in those with normo- and microalbuminuria, only 48% (22 of 44) of urine samples were positive for CN-1 (*p* < 0.0001). Likewise, CN-1 was detected in 88% of urine samples (15 of 17) from patients with nondiabetic CKD and macroalbuminuria as compared to 33% (3 of 9) of urine samples from patients with normo- and microalbuminuria (*p* = 0.007) (Table 1). Median urinary CN-1 concentrations in patients with T2DM and macroalbuminuria were significantly higher in comparison to those in T2DM patients with normo- and microalbuminuria (554 ng/ml [IQR 212-934 ng/ml] vs. 31 ng/ml [IQR 31-64 ng/ml], ACR > 300 vs. ACR ≤ 300 mg/g; *p* < 0.0001). This was also true for the nondiabetic CKD group (197 ng/ml [IQR 112-739] vs. 31 ng/ml [IQR 31-226 ng/ml], ACR > 300 vs. ACR ≤ 300 mg/g; *p* = 0.016) (Figure 1(a)).

Of note, the median urinary CN-1 concentrations of healthy individuals were significantly lower compared to those of T2DM patients with macroalbuminuria (*p* < 0.0001), and healthy individuals showed with borderline significance lower urinary CN-1 concentrations compared to the nondiabetic group with macroalbuminuria (*p* = 0.05) (data not shown).

In the overall population of patients (*n* = 111), a positive correlation between urinary CN-1 and ACR was found (*r* = 0.68, *p* < 0.0001) (Figure 1(b)). Patients with reduced renal function (eGFR < 60 ml/min/1.73m^2^) were found to have significantly higher urinary CN-1 concentrations compared to patients with preserved renal function (eGFR > 60 ml/min/1.73m^2^) (*p* = 0.015) (Figure 1(c)). When three eGFR strata (eGFR < 30, 30-60, and >60 ml/min1.73m^2^) were applied, urinary CN-1 concentrations differed between eGFR < 30 ml/min and >60 ml/min with borderline significance (*p* = 0.056) (data not shown).

### 3.3. Serum CN-1 Concentrations in T2DM and Nondiabetic CKD Patients and Healthy Individuals

Based on the findings that urinary CN-1 concentrations are increased in patients with macroalbuminuria regardless of the underlying renal disease and because our previous studies suggested low serum CN-1 concentrations to be present in T2DM patients with impaired renal function [33], we assessed if high urinary CN-1 concentrations were associated with decreased CN-1 levels in serum.

**Figure 2:**
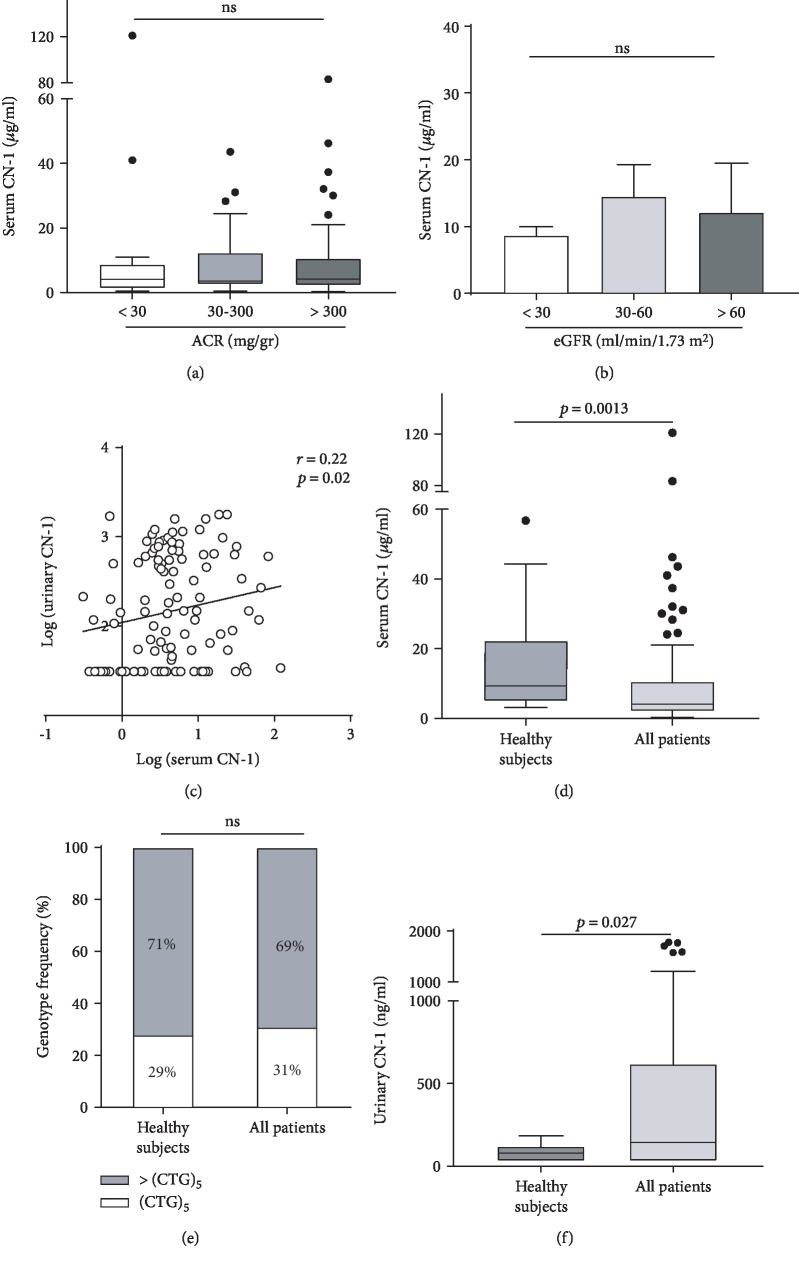
(a) Distribution of serum CN-1 concentrations in all the studied patients (*n* = 111) according to ACR in normoalbuminuria (*n* = 20), microalbuminuria (*n* = 33), and macroalbuminuria (*n* = 58). Boxes and whiskers represent the median and IQR. (b) According to eGFR in severely decreased (*n* = 72), mildly decreased (*n* = 23), and preserved eGFR (*n* = 16). (c) Spearman correlation of serum CN-1 [log (serum CN-1]) and urinary CN-1 [log (urinary CN-1)] concentrations in all patients (*n* = 111). (d) Patients (*n* = 104) display lower serum CN-1 concentrations in comparison to healthy subjects (*n* = 24). (e) Differences are not related to the distribution of the homozygous *CNDP1* (CTG)_5_ versus all other genotypes (>CTG)_5_ (*p* = ns). (f) Patients (*n* = 104) have higher urinary CN-1 levels compared to the healthy group (*n* = 24).

To test this, all patients were stratified on the basis of ACR in normo-, micro-, and macroalbuminuria (ACR < 30, 30-300, and >300 mg/g, respectively). As shown in Figure 2(a), there were no significant differences in serum CN-1 concentrations amongst these 3 groups. Serum CN-1 levels did not differ between patients with impaired and patients with preserved renal function (4.0 *μ*g/ml [IQR 2.5-10.53 *μ*g/ml] vs. 2.8 *μ*g/ml [IQR 0.97-10.43 *μ*g/ml]) for eGFR < 60 and eGFR > 60 ml/min/1.73m^2^, respectively (*p* = 0.25). Likewise, serum CN-1 concentrations were not different between patients with poor, moderate, or preserved renal function (Figure 2(b)), albeit the mean serum CN-1 concentration in patients with eGFR < 30 was the lowest (8.66 ± 1.33 vs. all other eGFR strata, *p* > 0.05). A weak but significant positive correlation between serum and urinary CN-1 concentrations was observed for the whole cohort of patients (*n* = 111) (*r* = 0.22, *p* = 0.02) (Figure 2(c)).

When patients were compared to the healthy group, serum CN-1 concentrations were significantly higher in healthy controls (9.4 *μ*g/ml [IQR 4.8-24.8 *μ*g/ml] vs. 4.1 *μ*g/ml [IQR 2.1-10.5 *μ*g/ml], *p* = 0.0013) (Figure 2(d)). This could not be explained by differences in genotype distribution of the homozygous *CNDP1* (CTG)_5_ genotype (Figure 2(e)) (*p* = 0.9), which is associated with low serum CN-1 levels, but may be related to the higher proportion of females included in the healthy control group (67% in the healthy vs. 35% in the disease group) (*p* = 0.0055).

The finding of low serum CN-1 levels in kidney disease patients was further investigated to control for potential confounders. Serum CN-1 concentration was set as the dependent variable, and a linear regression analysis including all participants (healthy subjects and kidney disease patients) (*n* = 128) was performed. In the crude model, the status of kidney disease patients was inversely correlated with low serum CN-1 concentrations (*β* = −0.258, *p* = 0.003). After adjustment for sex and age, the association of the kidney disease patient and serum CN-1 slightly weakened and was no longer significant (*β* = −0.219, *p* = 0.089) (model 1), but the association became uncovered again by further adjustment for urinary CN-1 concentrations (*β* = −0.281, *p* = 0.033) (model 2) (Supplementary [Supplementary-material supplementary-material-1]).

In line with this finding, higher urinary CN-1 concentrations were observed in all the kidney disease patients versus healthy subjects (143.7 ng/ml [IQR 31-619.6 ng/ml] vs. 79.13 ng/ml [IQR 31.7-121.7 ng/ml], *p* = 0.027; Figure 2(f)).

### 3.4. Influence of the CNDP1 Genotype on Serum and Urinary CN-1 Concentrations

**Figure 3:**
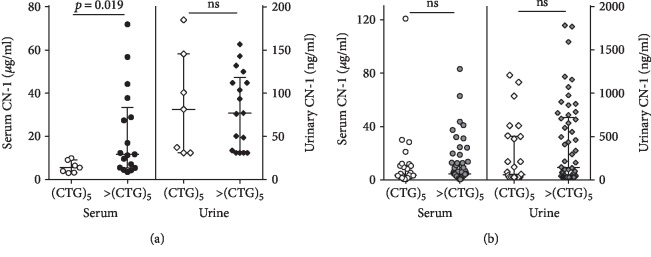
Distribution of serum CN-1 (left *y*-axis) and urinary CN-1 concentrations (right *y*-axis) according to the *CNDP1* (CTG)_5_ homozygous genotype versus other *CNDP1* genotypes (>CTG)_5_ in healthy subjects (*n* = 24) (a) and in patients with T2DM (*n* = 85) (b). Results are expressed for each individual patient. The lines represent the median and IQR. Note that the units on the left and right *y*-axes are expressed in *μ*g/ml and ng/ml, respectively.

We first assessed in healthy subjects if the homozygous *CNDP1* (CTG)_5_ genotype was associated with low serum and urinary CN-1 concentrations. Serum CN-1 concentrations were approximately 100-fold higher as compared to those found in spot urine (serum CN-1: median 9.4 *μ*g/ml [IQR 4.8-24.2 *μ*g/ml]; urinary CN-1: median 79 ng/ml [IQR 32-122 ng/ml]) (Figure 3(a)). While in serum *CNDP1* (CTG)_5_ homozygous individuals displayed significantly lower CN-1 concentrations as compared to all other genotypes (5.5 *μ*g/ml [IQR 3.2–9.1 *μ*g/ml] vs. 11.7 *μ*g/ml [IQR 5.5-33.3 *μ*g/ml], *p* = 0.019), this was not found for urinary CN-1 concentrations (81.3 ng/ml [IQR 31-146 ng/ml] vs. 77 ng/ml [IQR 33-118.5 ng/ml], *p* = 0.94) (Figure 3(a)). Neither in serum nor in urine of T2DM patients, differences in CN-1 concentrations were found between *CNDP1* (CTG)_5_ homozygous individuals and individuals carrying a different genotype (>CTG)_5_ (in serum: 3.5 *μ*g/ml [IQR 1.9-10.9] versus 4.4 *μ*g/ml [IQR 2.6-12.7] (*p* = 0.62) and in urine: 60 ng/ml [IQR 31-503.2] vs. 141.3 ng/ml [IQR 34.1-718.5] (*p* = 0.14)) (Figure 3(b)).

### 3.5. Serum CN-1 Concentrations and Albuminuria Are the Main Predictors of Urinary CN-1 in T2DM and Nondiabetic CKD Patients

To assess factors predicting urinary CN-1 concentrations, relevant kidney disease-associated variables, i.e., age, gender, baseline kidney disease (diabetic or nondiabetic CKD), renal replacement therapy, serum CN-1 concentrations, (CTG)_5_ homozygosity, eGFR, ACR, and residual diuresis, were selected as independent predictors of log-transformed urinary CN-1 concentrations [log (urinary CN-1 concentration)] (Table 2). In univariate analysis, the variables baseline kidney disease, renal replacement therapy, and residual diuresis did not reach the significance threshold of *p* < 0.25 and were excluded in the multivariate linear regression model (Table 2). ACR and serum CN-1 concentrations were found to be the strongest predictors of urinary CN-1 concentrations, all together explaining 47% of variation of log (urinary CN-1 concentration) (*R*^2^ = 0.47, *p* < 0.0001).

## 4. Discussion

Proteinuria, routinely assessed in patients with chronic kidney disease (CKD), is a well-established biomarker for the progression of renal function deterioration in patients with or without diabetes [34]. Assessment of proteinuria based on 24 h urine collection was previously considered the gold standard. Yet because of its inconvenience and collection inaccuracies [35, 36] in recent years, clinical practice guidelines [37] suggest the first void or spot urine albumin-to-creatinine ratio (ACR) to assess progression of CKD and treatment evaluation. As such, specimens of the first void or spot urine rather than 24 h urine are mostly collected in biorepositories of patients with CKD. Nonetheless, it should be emphasized that spot urine collections are liable to hourly variations in urinary protein and creatinine excretion and therefore may not match ACR if it had been determined based on a 24 h collection.

We have previously reported that CN-1 in 24 h urine of patients with T2DM is associated with renal function impairment and urinary albumin excretion rate [31]. In order to confirm these findings in larger cohorts where only spot urine is available, the present study was conducted as a feasibility study in a smaller group of CKD patients to assess the relation between CN-1 in spot urine, ACR, and renal function. Additionally, we tested the influence of serum CN-1 concentrations and the *CNDP1* genotype on urinary CN-1 expression. The main findings of our study are the following: Firstly, urinary CN-1 is more frequently detected and displays higher concentrations in patients with macroalbuminuria as compared to those with normo- or microalbuminuria. Secondly, a strong positive correlation between urinary CN-1 and ACR was found (*r* = 0.68, *p* < 0.0001). Thirdly, patients with severely to moderately impaired renal function (eGFR < 60 ml/min/1.73m^2^) displayed higher urinary CN-1 concentrations compared to patients with preserved renal function (147.5 vs. 33 ng/ml, *p* = 0.015). Fourthly, ACR and serum CN-1 concentrations are independent predictors of urinary CN-1, explaining together 47% of variation of urinary CN-1 concentrations (*R*^2^ = 0.47, *p* < 0.0001). These data are concordant with our previous results using 24 h urine samples and thus suggest that spot urine can be used for the assessment of CN-1 in biorepositories in which spot rather than 24 h urine samples were collected.

Based on the previous observation that T2DM patients with poor renal function have low serum CN-1 concentrations [33], we hypothesized that high urinary CN-1 concentrations in patients with poor renal function and albuminuria might be associated with low CN-1 concentrations in serum as a result of an impaired glomerular barrier. Although in the current study, mean serum CN-1 concentration in patients with poor renal function was numerically lower compared to that in patients with moderately impaired or preserved renal function, this did not reach statistical significance. It should be underscored however that in our previous study, only patients with T2DM were included of which 127 were assigned as having DN and 76% hereof were on renal replacement therapy [33]. This is in sharp contrast to the present study where only 85 patients with T2DM were included of which 38% required dialysis.

Because urinary CN-1 excretion is much higher in patients with impaired renal function but serum concentrations are not that much different, we presume that the total production of CN-1 goes up to compensate for losses, resulting in relatively similar serum concentrations despite the losses that occur into urine.

Nonetheless, the strong correlation between urinary CN-1 concentrations and albuminuria suggests that increasing CN-1 concentrations likely reflects impairment of the glomerular filtration barrier. This may also partly explain the positive correlation between serum CN-1 and urinary CN-1 concentrations in patients (Figure 2(c)) but not in healthy controls (data not shown), as in patients with an impaired glomerular filtration barrier, such a correlation would be expected. In line with this, in the multivariate regression model, serum CN-1 concentration was identified as an independent predictor of urinary CN-1 concentrations. Likewise, positive correlations between albumin synthesis and urinary albumin excretion have been reported in patients with macroalbuminuria [38].

Of note, high serum CN-1 concentrations were observed in the healthy subjects in comparison to the disease group (Figure 2(f)). Although it appeared that age and sex were influencing these differences, the uncovering effect by the addition of urinary CN-1 suggests that the association of kidney disease and low serum CN-1 concentrations is linked to urinary CN-1 concentrations (Supplementary [Supplementary-material supplementary-material-1]).

These observations support the assumption that serum CN-1 is lost into urine in patients with kidney disease, already in early stages of the disease, probably due to glomerular leakage or poor tubular reabsorption or a combination of both. In patients with kidney disease, serum CN-1 is replenished but not to physiological levels (Figure 2(d)).

Together, our findings reveal that serum CN-1 and urinary CN-1 under healthy and kidney disease conditions might follow opposite patterns: whereas healthy subjects have higher levels of serum CN-1 in comparison to kidney disease patients (Figure 2(d)), kidney disease patients excrete higher amounts of urinary CN-1 in comparison to the healthy individuals (Figure 2(f)).

Due to reduced diuresis and increased prevalence of protein energy wasting, correlations between serum and urinary CN-1 concentration might become blurred as renal function deterioration progresses. Additionally, low serum CN-1 concentrations have indeed been reported in pathologies characterized by a high catabolic state, e.g., neoplasia and liver cirrhosis [39–41], making protein energy wasting a likely factor influencing serum CN-1 concentrations in patients with low residual renal function. Alternatively, dialysis per se and metabolic acidosis, a common comorbidity of CKD, can promote enhanced protein breakdown and amino acid oxidation [42, 43].

Although renal CN-1 mRNA and protein expression has been reported [11, 13, 30], to the best of our knowledge, there are no studies that provide direct evidence for renal CN-1 secretion into the renal tubular lumen. Yet the lack of correlation between serum and urinary CN-1 concentrations in healthy individuals argues against CN-1 filtration and a free communication between the circulatory and urinary compartments and tempted us to presume that urinary CN-1 is a consequence of renal production and secretion. Nonetheless, it cannot be excluded that serum CN-1 partly exists in a monomeric conformation [44] and, based on a similar molecular weight and isoelectric point as albumin, might pass the filtration barrier.

It is also worthwhile to mention that in contrast to serum CN-1 concentrations, urinary CN-1 concentrations were not influenced by the *CNDP1* (CTG)*_n_* polymorphism. Since it has been shown that variations in the leucine repeat in the signal peptide of CN-1 affect CN-1 secretion [10], this would also be expected for renal CN-1 secretion. If however monomeric serum CN-1 is filtered, urinary CN-1 concentrations will correlate more with the fraction of monomeric CN-1 rather than the *CNDP1* genotype. Our in-house ELISA used for CN-1 detection cannot distinguish between CN-1 monomers and dimers, and the small amount of CN-1 as well as the presence of urea in the urine sample of healthy controls also hampers drawing firm conclusions from Western blotting experiments.

Although patients with low eGFR appeared to have higher urinary CN-1 concentrations compared to patients with preserved renal function (eGFR > 60 ml/min/1.73m^2^) and the univariate analysis revealed a significant negative correlation between eGFR and urinary CN-1, the subsequent multivariate regression model excluded eGFR as an independent explanatory factor for urinary CN-1. This could be explained by the following: (1) The currently studied population has considerable renal function impairment compared to our previous study. Thus, it might be that at late stages of the disease, eGFR influences urinary CN-1 concentrations to a lesser extent; (2) a possible collinearity between the variables eGFR and ACR may add similar information, and thus, only the variable with the strongest relationship is included in the final model [45]. Based on the latter, it can be concluded that for the group of patients, ACR is a stronger predictor of urinary CN-1 concentrations than eGFR. Noteworthy, the correlation coefficient between urinary CN-1 and albuminuria (ACR) in spot urine was higher, and the goodness of fit was even better than the one previously reported in the 24 h urine study (*r* = 0.68 and *R*^2^ = 0.47, *p* < 0.0001) versus (*r* = 0.59 and *R*^2^ = 0.37, *p* < 0.0001) [31].

The confirmation and reproducibility of the findings from the 24 h urine study in the present data suggest that the assessment of CN-1 in spot samples may be considered similarly reliable as in 24 h samples. This has already been suggested for the assessment of proteinuria [46]. Multivariate linear regression analysis revealed that albuminuria (ACR) and serum CN-1 can be considered independent predictors of urinary CN-1 in nondiabetic and diabetic patients with CKD. If we speculate that in this group of patients, the majority of detected CN-1 in urine originates from the circulation, these findings might imply that the concentration of CN-1 in urine is a function of two independent processes: on the one hand changes in glomerular permselectivity reflected by a strong positive correlation with albuminuria and on the other hand the ability of the liver to maintain CN-1 production.

Apart from the finding that urinary CN-1 likely reflects impairment of the glomerular filtration barrier, we propose that the additional value of urinary CN-1 as a potential biomarker resides in its enzymatic properties. Because the major CN-1 substrates carnosine and anserine are endowed with antioxidative and antiglycating properties, they may prevent/reduce oxidative or glycative tissue damage. If high urinary CN-1 is associated with low renal levels of these histidine-containing dipeptides (HCD), this may mirror the ability of the kidney to protect itself against noxious reactive oxygen or carbonyl species. The relation between high renal CN-1 activity and low renal HCD levels has been demonstrated in diabetic mice. CN-1 activity is increased by CN-1 carbonylation and possibly also by oxidative stress as indicated by the studies of Peters et al. and Bellia et al. [47–49].

### 4.1. Limitations of the Study

Even though the current study is in concordance with our previously published data, there are some limitations that need to be addressed. Firstly, the number of healthy controls is relatively small and healthy controls were not matched for gender and age with the patient cohort. Hence, conclusions based on any direct comparisons between patients and controls cannot be firmly drawn and should be taken with caution. Secondly, it would be useful to directly compare 24 h urine and spot urine of the same individual. We are aware that this would have been the most ideal situation; however, at the time of the study, 24 h urine samples were not available for most of the patients. The intention of this study was to assess the feasibility of meaningful measurement of urinary CN-1 concentrations in spot urine. Further epidemiological studies with a prospective design are warranted for evaluating the sensitivity, specificity, negative and positive predictive values of urinary CN-1 for CKD progression and decline in GFR.

## Figures and Tables

**Table 1:** Baseline characteristics of patients and healthy subjects.

	T2DM			CKD			Healthy subjects
Characteristics	ACR ≤ 300 mg/g (*n* = 44)	ACR > 300 mg/g (*n* = 41)	*p* value	ACR ≤ 300 mg/g (*n* = 9)	ACR > 300 mg/g (*n* = 17)	*p* value	*n* = 24
Demographic							
Male sex, *n* (%)	27 (61)	30 (73)	0.24	7 (78)	9 (53)	0.39	8 (33)
Age (years)	68.3 ± 15.1	62.4 ± 15	0.06	70 ± 16.3	65.8 ± 17.9	0.61	30.3 ± 9.3
Clinical							
Body mass index (kg/m^2^)	31.3 ± 7.3	30 ± 6.3	0.28	28 ± 3.9	29 ± 6.8	0.68	
Hba1c (%)	8.2 ± 2.4	7.6 ± 2.0	0.22	5.1 ± 0.5	5.4 ± 0.8	0.31	
Serum albumin (g/l)	28.7 ± 7.5	27.8 ± 6.6	0.56	29.2 ± 4.7	29 ± 5.9	0.93	
FPG (mg/dl)	154.5 ± 66.2	145 ± 64	0.50	96.3 ± 10.3	90 ± 11	0.15	
Systolic BP (mmHg)	134.3 ± 22.4	142.5 ± 16.8	0.06	131.2 ± 26.2	137.7 ± 19.4	0.48	
Diastolic BP (mmHg)	74.1 ± 13.1	75.3 ± 15.1	0.72	72 ± 13	76.3 ± 15.8	0.50	
Diabetic retinopathy, *n* (%)	19 (43)	31 (75)	0.004	—	—	—	
Kidney function							
Serum creatinine (mg/dl)	2.5 ± 1.7	4.7 ± 3.3	0.002	3.8 ± 2.0	4.4 ± 3.1	0.58	
eGFR (ml/min/1.73m^2^)	33 [17–60]	13 [7.5–30]	0.0005	12.6 [8.3–49.4]	11.9 [7.9–32]	0.80	
Albuminuria ACR (mg/g)	49.5 [13.3–112]	1498 [580–3261]	<0.0001	127 [31–207.5]	1150 [641.5–2099]	<0.0001	
Dialysis *n* (%)	13 (30)	20 (49)	0.08	4 (44)	9 (53)	0.68	
Hemodialysis, *n* (%)	12	18		4	9		
Peritoneal dialysis, *n* (%)	1	2		—	—		
Pharmacological management							
ACEi/ARB, *n* (%)	25 (57)	25 (61)	0.70	4 (44)	9 (53)	0.68	
Diuretic, *n* (%)	30 (68)	29 (71)	0.80	7 (78)	12 (71)	0.69	
CN-1 metabolism							
CN-1 in serum (*μ*g/ml)	3.9 [2.1–12]	4.6 [2.6–12.2]	0.74	3.0 [1–4.6]	3.9 [0.9–6.3]	0.70	9.4 [4.8–24.8]
Urinary CN-1 detection, *n* (%)	22 (48)	39 (95)	<0.0001	3 (33)	15 (88)	0.007	18 (75)
Homozygous (CTG)_5_, *n* (%)^∗^	15 (37)	11 (28)	0.48	3 (43)	3 (18)	0.31	7 (29)

Baseline characteristics of T2DM and nondiabetic CKD patients related to albuminuria (ACR) and healthy subjects. Data are shown as mean ± standard deviation, median [IQR], or absolute number (proportion). *p* values were calculated using the two-tailed unpaired *t*-test, Mann-Whitney test, or Fisher's test. T2DM: type 2 diabetes patients; eGRF: estimated glomerular filtration rate using the CKD-EPI formula; HbA1c: hemoglobin A1c; ACEi/ARB: angiotensin-converting enzyme inhibitor/angiotensin receptor blocker. ^∗^(CTG)*_n_* genotype data were missing in 7 out of 111 patients.

**Table 2:** Summary of univariate and multivariate regression analyses predicting urinary CN-1 concentrations in T2DM and nondiabetic CKD patients (*n* = 104).

	Univariate analysis				Multivariate analysis			
Variable	Coefficient (*β*)	95% CI		*p* value	Coefficient (*β*)	95% CI		*p* value
Age (years)	-0.007	-0.014	0.001	0.092	0.002	-0.005	0.010	0.56
Male sex	0.17	-0.08	0.42	0.18	0.14	-0.044	0.33	0.13
Nondiabetic kidney disease	0.075	-0.208	0.36	0.60	—	—		—
On renal replacement therapy	-0.095	-0.34	0.15	0.43	—	—		—
CNDP1 (CTG)_5_ homozygosity	-0.500	-0.43	0.08	0.17	-0.033	-0.23	0.16	0.74
Log (serum CN-1)	0.195	-0.012	0.40	0.065	0.21	0.053	0.36	0.009
Log (ACR)	0.47	0.37	0.57	<0.0001	0.48	0.37	0.60	<0.0001
eGFR (ml/min/1.73 m^2^)	-0.009	-0.009	-0.0004	0.032	0.001	-0.003	0.005	0.66

Univariate and multivariate regression models with log-transformed urinary CN-1 as the dependent variable. Goodness of fit of the multivariate regression full model: adjusted *R*^2^ = 0.47; *p* < 0.0001.

## Data Availability

The clinical data used to support the findings of this study are restricted by the Ethical Commission II from the University Medical Center Mannheim, University of Heidelberg, in order to protect patient privacy. Data are available from B.A.Y. (benito.yard@medma.uni-heidelberg.de) for researchers who meet the criteria for access to confidential data.

## Supplementary Materials

Supplementary Materials Supplementary Table 1. Regression analysis for serum CN-1 concentrations as the independent variable with the studied groups (healthy subjects or kidney disease patients) (*n* = 128) as the dependent variables. Serum CN-1 was log-transformed for analyses. Coefficients are provided as standardized *β* values. Kidney disease, crude association; model 1, adjusted for sex and age; and model 2, additionally adjusted for log urinary CN-1.
