# Supplementary material for: Serum Carnosinase-1 and Albuminuria Rather than the CNDP1 Genotype Correlate with Urinary Carnosinase-1 in Diabetic and Nondiabetic Patients with Chronic Kidney Disease
Source: J Diabetes Res. 2019 Dec 24;2019:6850628. doi: 10.1155/2019/6850628 (PMC6948305; doi:10.1155/2019/6850628)
Supplement: Supplementary Materials — Supplementary Table 1. Regression analysis for serum CN-1 concentrations as the independent variable with the studied groups (healthy subjects or kidney disease patients) (n = 128) as the dependent variables. Serum CN-1 was log-transformed for analyses. Coefficients are provided as standardized β values. Kidney disease, crude association; model 1, adjusted for sex and age; and model 2, additionally adjusted for log urinary CN-1. [file 6850628.f1.pdf]

**Supplementary table 1**. **Regression analysis for serum CN-1 concentrations as the dependent variable with the studied groups (healthy subject or kidney-disease patient) (n=128) as the independent variable**

|  | Coefficient (β) | 95% CI | *p-* value |
| --- | --- | --- | --- |
| Kidney disease | -0.258 | - 0.613 - - 0.125 | 0.003 |
| Model 1 | -0.219 | - 0.676 - 0.049 | 0.089 |
| Model 2 | -0.281 | - 0.771 - - 0.033 | 0.033 |

Serum CN-1 was log-transformed for analyses. Coefficients are provided as standardized β values. Kidney disease, crude association; Model 1, adjusted for sex and age; Model 2, additionally adjusted for log urinary CN-1.
